# Sporadic Dyschromatosis Universalis Hereditaria: A Second Case Report From Iran

**DOI:** 10.7759/cureus.16511

**Published:** 2021-07-20

**Authors:** Sasan Dogohar, Pedram Alirezaei, Hamidreza Ghasemi Basir, Mohammad Jamshidi, Farshid Etaee

**Affiliations:** 1 Psoriasis Research Center, Hamadan University of Medical Sciences, Hamadan, IRN; 2 Department of Pathology, Hamadan University of Medical Sciences, Hamadan, IRN; 3 Internal Medicine, Yale University, New Haven, USA; 4 Internal Medicine, Texas Tech University Health Sciences Center at Amarillo, Amarillo, USA

**Keywords:** dyschromatosis universalis hereditaria, duh, dermatology case report, genetic disorders, pigmentary disorders

## Abstract

Dyschromatosis universalis hereditaria (DUH) is a rare pigmentary genodermatosis mostly reported from Japan. It is usually characterized by widespread hyper/hypopigmented macules all over the body. Here, we report the case of a patient from Iran who presented with disseminated hyper and hypopigmented lesions over the trunk, neck, and extremities since the age of eight. To the best of our knowledge, to date, there has been only one reported case of DUH from Iran.

## Introduction

Dyschromatosis universalis hereditaria (DUH) is a rare autosomal dominant or sometimes autosomal recessive genodermatosis with generalized mottled hyperpigmented and hypopigmented macules. DUH was first reported by Ichikawa et al. in 1933 [1]. Since then, most cases have been described from Japan. Additional cases have been published from Europe, South America, India, China, Iraq, South Africa, Saudi Arabia, Tunisia, and Iran. It usually appears in early childhood and persists into adulthood. There may be some associated conditions like neurologic, ocular, and metabolic disorders. There are similar diagnoses that can often be differentiated with history, physical examination, and skin biopsy. In rare circumstances, the lesions may be observed over the palms, soles, and mucous membranes. The involvement of nails has also been described [2]. DUH mostly presents before the age of six. Nonetheless, the late-onset disease may also occur. Here, we describe the second case of DUH in Iran.

## Case presentation

**Figure 1:**
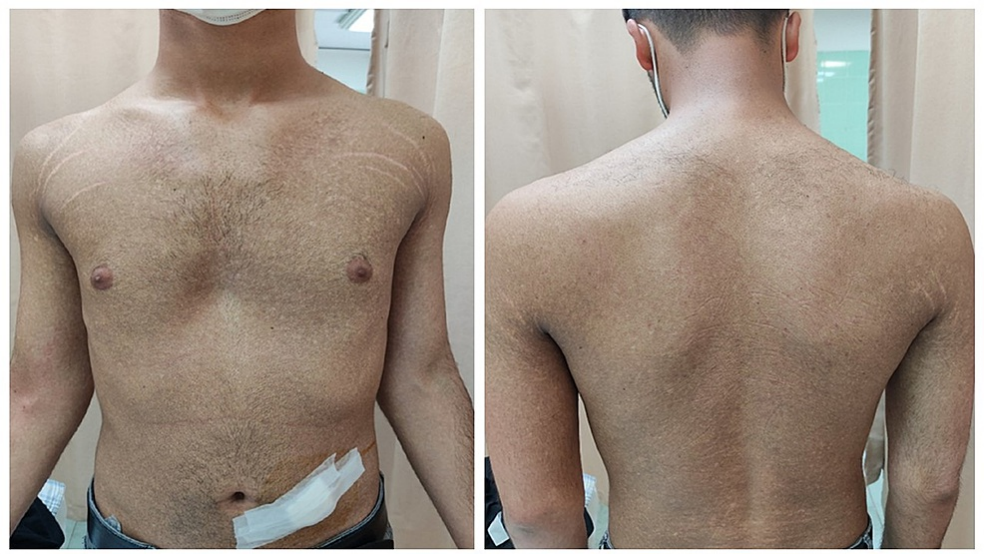
Diffused mottled hyper/hypopigmented macules over the trunk.

**Figure 2:**
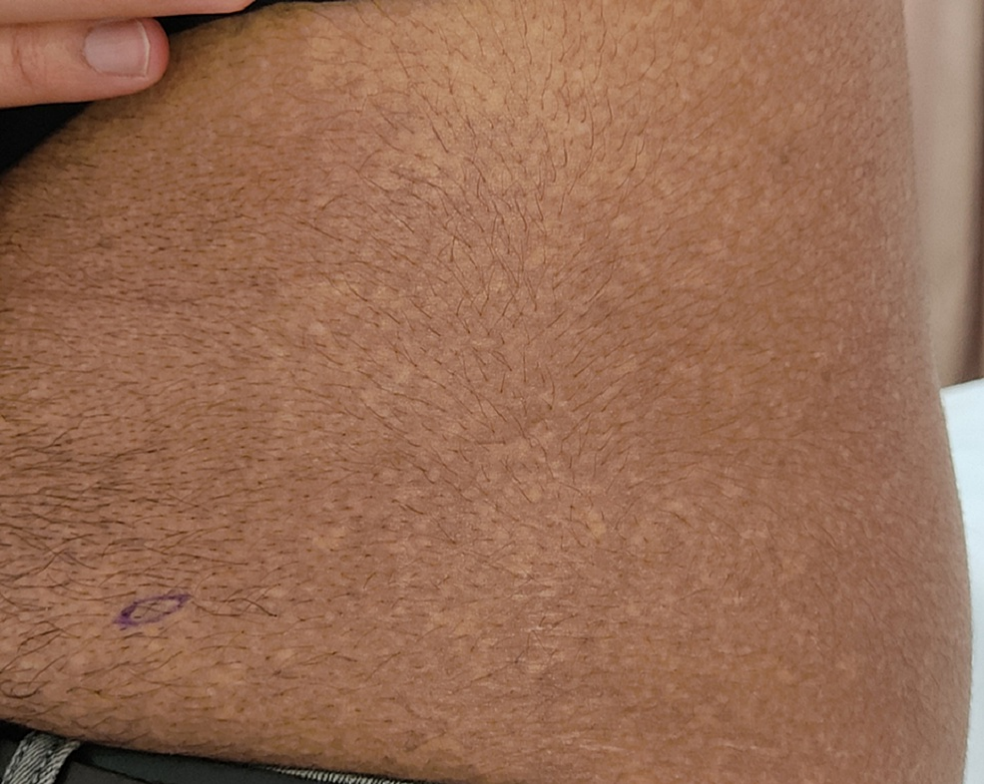
Close-up view of the patient.

A 20-year-old male presented to our outpatient dermatology clinic with a complaint of multiple disseminated hyper/hypopigmented lesions all over his body since the age of eight. The lesions started from the trunk and proximal extremities and gradually increased in number. The lesions were distributed all over the trunk, extremities, and neck, sparing the face, palms, and soles without the involvement of hair, nail, and mucosae. There was no skin scarring or telangiectasia (Figures 1, 2).

There was no significant past medical history including photophobia, seizure, diabetes, or any topical or systemic drug usage. He was born of nonconsanguineous parents, and none of his family members was affected, suggesting a sporadic inheritance.

**Figure 3:**
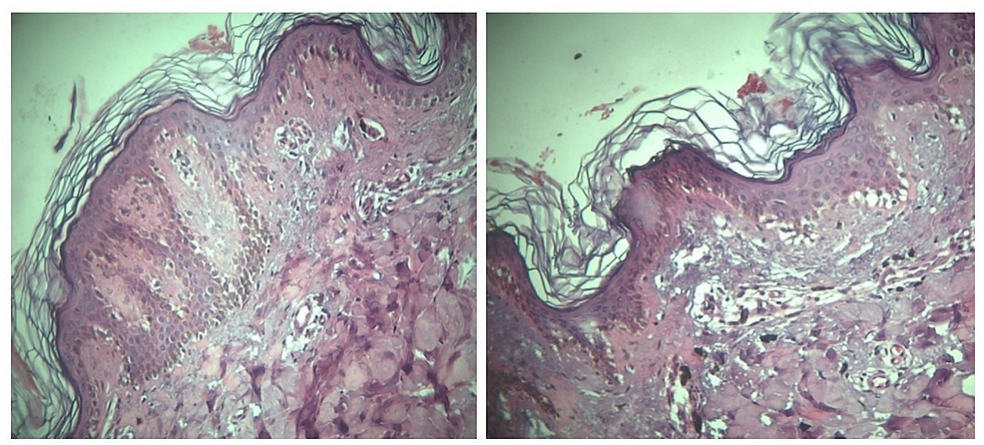
Mild hyperkeratosis, basal layer hyperpigmentation, obvious melanin incontinence, and scattered lymphocytic infiltration in the upper dermis.

We performed an elliptical skin biopsy from the abdomen including hyper/hypopigmented macules. The pathology sections showed skin tissue with mild hyperkeratosis, basal layer hyperpigmentation, obvious melanin incontinence, and scattered lymphocytic infiltration in the upper dermis (Figure 3).

Based on the clinicopathological correlation, the diagnosis of DUH was finally made. The patient was informed of the benign course of the disease and the possible unsatisfactory response to treatment options.

## Discussion

DUH is a rare pigmentary genodermatosis characterized by asymptomatic numerous small hyper/hypopigmented macules distributed mostly over the trunk and extremities [2]. Although DUH often presents during early childhood, it can present at birth or start later in adulthood. In all reported cases, spontaneous regression has not been reported. The palms and soles are usually spared, and facial involvement is present in about 50% of cases [3]. It may rarely involve hair, nail, and mucosae.

In some studies, DUH has been considered to be a generalized form of a disease spectrum that includes DUH, dyschromatosis symmetrica hereditaria (confined to extremities), and a segmental form called unilateral dermatomal pigmentary dermatosis [4]. Other differential diagnoses of DUH include amyloidosis cutis dyschromica, xeroderma pigmentosum, dermatopathia pigmentosa reticularis, Naegeli-Franceschetti-Jadassohn syndrome, and dyskeratosis congenita [5]. In particular, xeroderma pigmentosum should be considered and ruled out because both diseases can involve similar body areas. However, in our case, mostly non-sun-exposed areas were involved and no atrophy, scarring, or telangiectasia were seen, which is in contrast to xeroderma pigmentosum. In amyloidosis cutis dyschromica, amyloid deposits are present within the papillary dermis just below the epidermis. In dermatopathia pigmentosa reticularis, a patient may present with noncicatricial alopecia and onychodystrophy. In Naegeli-Franceschetti-Jadassohn syndrome, patients may present with a reticulate pattern of skin hyperpigmentation, palmoplantar keratoderma, abnormal sweating, and other subtle developmental anomalies of teeth, hair, and skin. Finally, dyskeratosis congenita is a rare genetic form of bone marrow failure, which is the inability of the marrow to produce sufficient blood cells, in addition to skin findings.

Previously, it was believed that DUH occurs only in the Japanese population. However, lately, it has been repeatedly described in other populations and regions as well [6]. In Iran, it was first reported by Amirnia et al. in 2008 [7]. The literature review confirms that our case is the second case of this rare disease from Iran.

DUH is mostly an autosomal dominant or sometimes autosomal recessive genodermatosis, but sporadic forms have also been reported. There are three subtypes of DUH with specific genetic patterns, including DUH1 (6q24.2-q25.2), DUH2 (12q21-q23), and DUH3 (2q35). DUH is a disease of melanin content rather than melanocyte number, and histology shows a high melanin concentration in hyperpigmented and a low concentration in hypopigmented lesions [8].

Although it is generally asymptomatic, systemic abnormalities have been reported in DUH, including neurologic problems, seizure, mental retardation, hearing loss, diabetes mellitus, ocular abnormalities, photosensitivity, tryptophan metabolism [6], and hypospadias [8]. Our patient denied any history of these conditions.

As there is no satisfactory treatment, we decided to manage our patient conservatively. However, a case report from India showed a moderate response to narrowband UVB phototherapy [4]. In a study, Li et al. treated a 5 cm × 1 cm area of lentigines on the forearm in a patient with DUH with a 755-nm Q-switched alexandrite laser [9].

## Conclusions

DUH is a rare pigmentary genodermatosis mostly reported from Japan. It should be considered in the differential diagnosis of all cases manifesting with mixed hyper and hypopigmented macules, and biopsy specimens should be obtained to confirm the diagnosis. DUH is often an autosomal dominant or sometimes an autosomal recessive genodermatosis; however, sporadic forms have also been described. To date, there is no satisfactory treatment for this dermatologic condition. Therefore, early diagnosis can prevent the application of ineffective treatments. To the best of our knowledge, this is the second report of DUH from Iran 13 years after the first report was published.
